# Cardiac Fibrosis: Key Role of Integrins in Cardiac Homeostasis and Remodeling

**DOI:** 10.3390/cells10040770

**Published:** 2021-03-31

**Authors:** Patrick B. Meagher, Xavier Alexander Lee, Joseph Lee, Aylin Visram, Mark K. Friedberg, Kim A. Connelly

**Affiliations:** 1 Keenan Research Centre, Li Ka Shing Knowledge Institute, St. Michael’s Hospital, Toronto, ON M5B 1W8, Canada; patrick.meagher@mail.utoronto.ca (P.B.M.); xavier.lee@mail.utoronto.ca (X.A.L.); josephjh.lee@mail.utoronto.ca (J.L.); aylin.visram@mail.utoronto.ca (A.V.); 2 Department of Physiology, University of Toronto, Toronto, ON M5S 1A8, Canada; mark.friedberg@sickkids.ca; 3 Institute of Medical Science, University of Toronto, Toronto, ON M5S 1A8, Canada; 4 Labatt Family Heart Center and Department of Paediatrics, Hospital for Sick Children, Toronto, ON M5G 1X8, Canada

**Keywords:** cardiac fibrosis, integrins, fibroblasts, myofibroblasts, left ventricle, right ventricle, mechano-sensing

## Abstract

Cardiac fibrosis is a common finding that is associated with the progression of heart failure (HF) and impacts all chambers of the heart. Despite intense research, the treatment of HF has primarily focused upon strategies to prevent cardiomyocyte remodeling, and there are no targeted antifibrotic strategies available to reverse cardiac fibrosis. Cardiac fibrosis is defined as an accumulation of extracellular matrix (ECM) proteins which stiffen the myocardium resulting in the deterioration cardiac function. This occurs in response to a wide range of mechanical and biochemical signals. Integrins are transmembrane cell adhesion receptors, that integrate signaling between cardiac fibroblasts and cardiomyocytes with the ECM by the communication of mechanical stress signals. Integrins play an important role in the development of pathological ECM deposition. This review will discuss the role of integrins in mechano-transduced cardiac fibrosis in response to disease throughout the myocardium. This review will also demonstrate the important role of integrins as both initiators of the fibrotic response, and modulators of fibrosis through their effect on cardiac fibroblast physiology across the various heart chambers.

## 1. Introduction

Heart Failure (HF) is a significant clinical problem with ~6 million individuals suffering from HF within the United States between 2015 and 2018 [1]. In response to toxic stimuli, the heart undergoes a response known as remodeling; characterized by cardiomyocyte (CM) hypertrophy, fibrosis along with a wide array of electrical and molecular based changes [2,3,4]. Cardiac fibrosis a key feature of the remodeling response, and is defined as the accumulation of excessive amounts of extracellular matrix (ECM) proteins such as collagen and fibronectin, in response to injuries such as myocardial infarction (MI), pressure overload (PO), or during the progression of inflammatory disorders such as diabetes [3,5,6]. Cardiac Fibroblasts (CFs) within and around injured areas become activated, and transform into myofibroblasts [7]. Myofibroblasts possess both contractile and synthetic characteristics, and are capable of secreting inflammatory cytokines as well as ECM components such as collagen [8]. In the heart, the transformation of CF to myofibroblast is associated with the production of fibrillar collagen types I and III, of which type I collagen remains the most abundant [9]. While the process of tissue repair is an important short-term adaptation to tissue injury, continual long-term production of ECM proteins results in a stiff, non-compliant myocardium which leads to cardiac dysfunction and culminates in HF [3,6,10,11]. Further, the increased production and accumulation of collagen along with the increased myofibroblast number leads to impaired mechano-electric coupling of CMs [12,13]. Finally, active myofibroblasts continue to secret proinflammatory cytokines further leading to CM hypertrophy, necrosis and prompting additional replacement fibrosis. This is best described by a positive feedback loop whereby injury leads to fibrosis, resulting in reduced chamber compliance and impaired cardiac function, which further activates cytokines leading to ongoing collagen production. One of the established contributors to cardiac fibrosis is mechanical stress. Since fibroblasts are sensitive to their mechanical microenvironment, increases in ECM tension observed during pathological remodeling further promotes endogenous CF differentiation to myofibroblasts. This further augments the positive feedback loop in that a stiffer matrix stimulates more collagen production. The molecular mechanisms involved in myofibroblast differentiation remains an active area of research, and a crucial part of the cell’s mechanosensory capability are the integrin family of proteins.

Integrins are family of heterodimeric transmembrane cell-adhesion molecules comprised of α and β receptor subunits [14,15,16]. There are to date, eighteen α subunits and eight β subunits which comprise twenty-four unique heterodimers. Integrins have two major functions; the first is to bind the ECM to the cell, and the second is to regulate cell-ECM signaling. The distinct role that each integrin heterodimer plays are dependent upon its composition of ⍺ and β subunits. These roles can include cell adhesion, migration and extravasation [14,16,17]. Importantly, integrin’s respond to ligand binding and are therefore classified by ligand specificity. These include arginine-glycine-aspartate (RGD)-binding receptors, leukocyte-specific receptors, laminin receptors and collagen binding receptors [14,16,18]. Moreover, specific integrins may mediate cell–cell adhesion and potentially communication [16]. Activation of integrins following ECM ligand binding (‘outside-in’ signaling), initiates downstream signaling mechanisms including A kinases, small GTPases and proteins that modulate ECM composition [17,19,20,21]. The affinity of an integrin to its ligand is enhanced in response to intracellular signaling (‘inside-out’ signaling) derived from activated G-protein or via coupling with other receptors [21]. Further, inside-out signaling mechanisms enable the docking of focal adhesions [22]. In this review we will explore the most recent research on the interaction of integrins and collagen in the heart and how these interactions are altered during disease.

## 2. Integrins in the Heart

The composition of most tissues in the body can be classified as cellular and extracellular components. The heart is composed of CMs, CFs, smooth muscle cells, and endothelial cells among others (e.g., pericytes and immune cells) [16]. The extracellular component, also known as the ECM of the heart contains a variety of molecules including hyaluronan, fibronectin, proteoglycans, laminin, and collagens [16,23]. These extracellular molecules communicate with the cells of the heart to help control cell growth, survival, differentiation, migration, and other cell processes [24]. This communication is mediated through several different mechanisms, but one of particular importance is through the cell-surface receptors like integrins [16].

**Figure 1:**
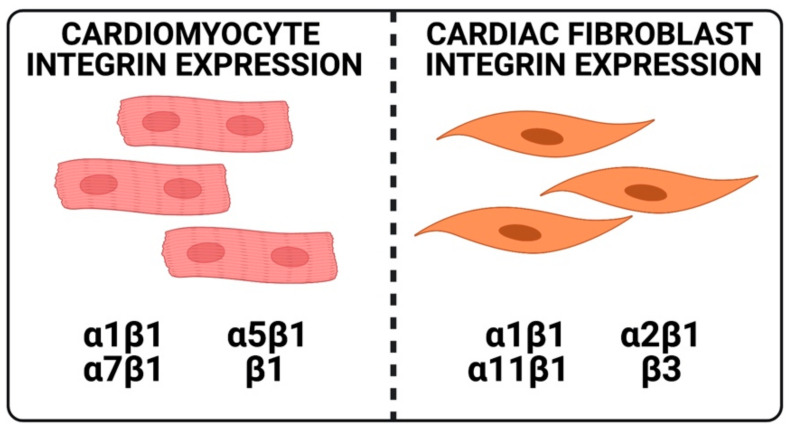
Integrin expression in healthy adult cardiomyocyte and cardiac fibroblast. In the healthy heart, adult cardiomyocytes have been shown to express ⍺1β1, ⍺5β1, ⍺7β1, and β1 integrins. In the adult fibroblast, integrins have been shown to express ⍺1β1, ⍺2β1, ⍺11β1, and β3 integrins.

In the healthy heart, CFs have been shown to express ⍺1β1 [25,26], ⍺2β1 [25,26], ⍺11β1 [27], and β3 integrins [28], CMs have been shown to express ⍺1β1 [29], ⍺5β1 [30], ⍺7β1 [15], and β1 integrins (Figure 1).

These integrins bind to a variety of ligands and ECM components. ⍺1β1, ⍺2β1, and ⍺11β1 are collagen-binding integrins [31], binding to collagens types I, IV, and IX [16,32,33,34]. These integrins differ in their preferential binding between collagens types I and IV. Between these two collagen types, ⍺1β1 preferentially bind to collagen type IV [18,32,33], while ⍺2β1 and ⍺11β1 preferentially bind to collagen type I [18,32,33,34]. Fibrillar collagens are the most abundant proteins within the cardiac ECM [35,36]. The primary role of collagen within the heart is to provide the structural lattice for CMs along with, stiffness to the myocardial wall and aid in force transduction [37,38]. Therefore, collagen synthesis and degradation is highly regulated within the myocardium and minor alterations to the collagen structure may significantly affect myocardial force production and ultimately cardiac function [39,40]. Further, collagen binding integrins play a relatively minor role in normal physiology but, have significant roles in development and organ fibrosis [18]. In addition to collagen, ⍺1β1 and ⍺2β1, along with ⍺7β1 [15,41] bind to laminin, another ECM molecule. The β3 [28] and ⍺5β1 [42,43] integrins have been shown to bind to Arg-Gly-Asp (RGD) motifs. The ⍺5β1 integrin has been shown to be the one predominantly expressed by the neonatal CM, while the ⍺7β1 integrin is predominantly expressed by the adult CM [15]. The ⍺vβ5 integrin, similar to the β3 and ⍺5β1 integrins, binds to RGD motifs [44], and is normally highly expressed during cardiac development, rather than in the adult heart [45]. ⍺3β1 is an integrin that binds to a variety of molecules, including fibronectin and laminin [46]. 

Fibronectin is a multifunctional, soluble, secreted glycoprotein produced by CFs, myofibroblasts, and endothelial cells [47]. Much like collagen, the organization of a fibronectin matrix is essential to cell migration in embryogenesis, and wound healing. To this end, integrin binding is required to scaffold fibronectin to the actin cytoskeleton and promote fibronectin fibrillogenesis. It has long been established that the primary fibronectin-binding integrin is the α5β1 integrin and knockout animal studies have been used to demonstrate a compensatory ability of the αVβ3 to mediate fibronectin attachment in the absence of α5β1 [48]. Attachment of these integrins to fibronectin mediate its alignment to actin stress fibers via RhoA-dependent mechanisms [49]. Recent work on fibronectin in the cardiac system has revealed its crucial role in embryological heart development. Work by Mittal et al. in fibronectin-null and α5-null mouse embryos displayed aberrations in fibroblast growth factor 8 (Fgf8) signaling without alterations in Fgf8 mRNA. The group also demonstrated that fibronectin adhesion was necessary to in vitro Fgf8 signaling, which purports a crucial role for α5β1 integrin in the adhesion of cardiac precursors to the fibronectin matrix and furthermore, mediating morphogenesis of the fetal heart [30].

## 3. Normal Functions of Integrins in CFs

The collagen-binding integrins ⍺1β1, ⍺2β1, ⍺11β1 mediate numerous functions in CFs in addition to collagen binding. The ⍺1β1 play important roles in the regulation of collagen synthesis [50,51] and fibroblast proliferation [52], ultimately contributing to the formation of new ECM. While ⍺2β1 alone functions in the induction of matrix metalloproteinases [50], both ⍺2β1 and ⍺11β1 share functions in contraction of collagen lattices [34,50,53,54], cell migration [34,55], and collagen fibril formation [56]. β3 integrins have been shown to contribute to cell spreading, proliferation, and migration [28].

## 4. Normal Functions of Integrins in CMs

CMs are responsible for the synchronous generation of force and relaxation that are the fundamental properties of heart function. To this end, CMs have a highly organized cellular structure, and must communicate with adjacent cells [57]. The integrins expressed in CMs support proper cell shape and proper cell–cell communication. ⍺1β1 is important in development and maintenance of cell morphology [29] while ⍺5β1 mediates organization of the gap junction protein, connexin 43 [58], which connects adjacent cells and allows for free movement of ions, metabolites, second messengers, and other small molecules between cells. The ⍺7β1 integrin has been shown to interact with laminin in order to promote cell adhesion and motility in myoblasts, the precursors to striated muscle cells (such as CMs) [59]. The β1 integrin possesses four isoforms, two of which are expressed in CMs, namely β1A and β1D. The β1A isoform is expressed predominantly in the developing heart, as opposed to β1D which is the isoform expressed in the adult heart. β1D functions in adhering the CM to the ECM [15] and helps maintain proper mechano-sensing in CMs [60]. Therefore, integrins act to coordinate and control force sensing, providing a mechanism by which the heart can sense and respond to the various mechanical stressors placed upon the heart during both health and disease in order to maintain appropriate cardiac function.

## 5. Integrins in the Diseased Heart

**Figure 2:**
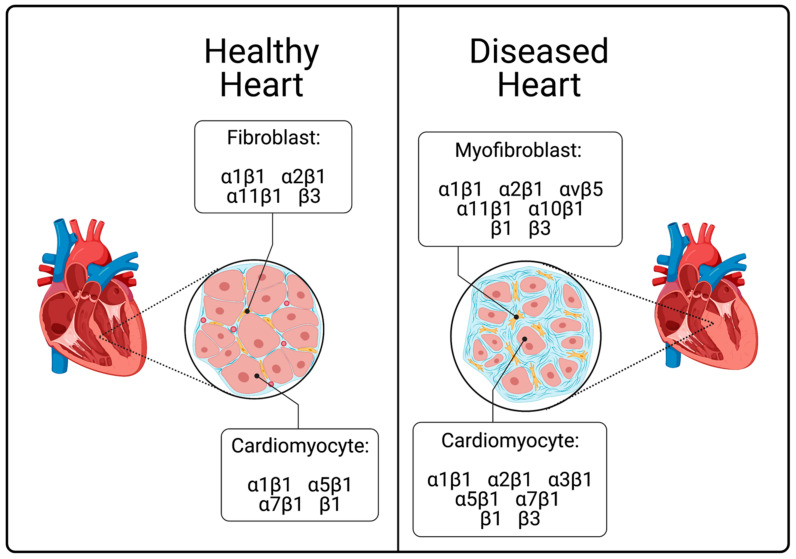
Summary of integrin expression in health and disease. The diseased heart possesses a higher proportion of myofibroblasts than does the healthy heart. In the diseased heart, myofibroblasts show modified expression of the α1β1, α2β1, α11β1, and β3 integrins, as well as non-endogenous expression of the αvβ5, α10β1, and β1 integrins. In the diseased heart, cardiomyocytes show modified expression of the ⍺1β1, α5β1, α7β1, and β1 integrins, as well as non-endogenous expression of the α2β1, α3β1, and β3 integrins.

In the diseased heart, the expression and function of integrins is altered in order to account for abnormal stress signals, leading to alterations in CFs, the ECM and CMs. Myocardial stress induced integrin signaling may result in the activation of myofibroblasts (e.g., fibrosis, or “the excessive deposition of collagen”) [61] or the development of CM hypertrophy [62]. CF expression of ⍺1β1 [16,25,26], ⍺2β1 [16,25,26], ⍺10β1 [63], ⍺11β1 [16,64] and β3 [65] integrins are modified in response to myocardial stress in both heath (e.g., exercise) and disease (e.g., MI), and the ⍺vβ5 [66], β1 [65] integrins become upregulated (Figure 2). CM expression of the ⍺1β1 [29], ⍺5β1 [30,67], ⍺7β1 [68], β1 [46,69] integrins are upregulated, and the ⍺2β1 [16], ⍺3β1 [67], β3 [45] integrins become expressed (Figure 2). 

The modification of integrin expression impacts the force sensing capabilities of the cell and alters signaling mechanisms within the cell (i.e., outside in signaling), as described below.

## 6. The Importance of Actin Binding Elements and Integrin Related Kinases

Since integrins do not possess intrinsic enzymatic or actin-binding activity, adaptor proteins are required to propagate signals from the ECM to the cell and vice versa. These adaptor proteins connect cytoplasmic tails of integrin α and β subunits to the actin cytoskeleton or other signaling molecules and mediate the activation of downstream pathways. The following section will outline the functionality of the main integrin-related proteins: Talin, Paxillin, Kindlin, Focal Adhesion Kinase (FAK) and Integrin-Linked Kinase (ILK) as they have been shown to be involved directly in integrin induced fibrosis.

## 7. Talin

**Figure 3:**
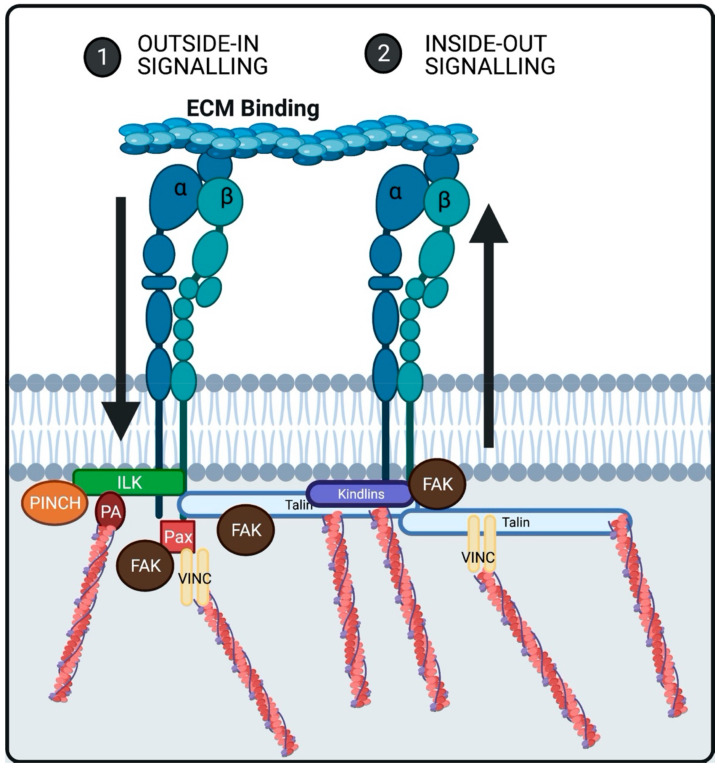
Integrins bind with the ECM followed by the binding of integrin associated proteins such as Talin, Kindlins, Paxillin (Pax), Focal adhesion Kinase (FAK) and Integrin Linked Kinase (ILK). Where the activation of Talin results in binding of the actin cytoskeleton directly or via vinculin (VINC) as well as, the activation of other Integrins through co-operation with Kindlin’s (Inside-out). Moreover, Talin binding results in the activation of FAK Paxillin binding to integrin results in FAK activation and actin binding via VINC. ILK binding to integrin results in cytoskeleton binding through Parvin (PA). Finally, FAK activation can occur via direct binding to integrins.

Talin is an actin-binding protein which binds either directly or indirectly through vinculin (Figure 3) [17,70]. Talin has broad effects upon integrin function, transducing signals across integrins both inside-out and outside-in. Talin modulates integrin organization through the actin network and focal adhesions composition [22,71,72]. Talin binding is initiated following ECM bound integrins recruiting talin to bind to the tails of β subunits such as integrin β1. Talin can facilitate non-ECM bound integrins to bind to their respective ligands which is thought to be dependent on FAK signaling [22,72] (Figure 3). Further, talin has two integrin binding sites, three actin binding sites and can dimerize. Thus, talin has the potential to bind four integrin heterodimers and multiple actin filaments [22]. The knockout (KO) of talin-1 within the heart leads to reduced hypertrophy and fibrosis in response to PO attributed to attenuated ERK1/2, p38 and Akt responses [73]. Manso et al., further investigated the roles of talin-1 and 2 demonstrating that talin2 KO surprisingly does not affect cardiac function or structure due to a compensatory upregulation of talin1. Finally, the KO of both talin1 and 2 leads to cardiac dysfunction and mortality by 6 months of age in mice [74]. 

## 8. Paxillin

Paxillin is an adapter protein that has been shown to be involved in integrin mediated cell adhesion to the ECM. Paxillin is a phosphotyrosine-containing protein that has been implicated in a number of signaling pathways due to the various adapter proteins and kinases in which it binds to, including vinculin (Figure 3), actopaxin, FAK and ILK [72]. Due to the wide range of protein interactions, it has been implicated in multiple mechanisms. Paxillin has been shown to directly bind to β1, β3 and ⍺4 integrin [75]. Paxillin is essential for heart function as paxillin deficiency leads to destabilization of binding partners such as FAK, resulting in degradation of vinculin and leading to HF [76].

## 9. Kindlin

Kindlins are a family of structurally related multidomain proteins consisting of three distinct subtypes, all of which bind to the cytoplasmic tail of β integrins. The assembly of focal adhesions requires the binding of kindlins and kindlins are important co-activators of integrins via number of mechanisms including the creation of a complex with talin and β1 integrin [72] (Figure 3). Various subtypes of kindlins exist: kindlin 1, 2 and 3 have different expression patterns with kindlin 1 and 2 expression ubiquitous across murine and human tissues where-as, kindlin-3 is restricted to hematopoietic tissues. Importantly all three kindlin isoforms bind to the collagen binding β1 integrin. The loss of kindlin 1 or 2 has been demonstrated to impair β1 integrin activation [77]. Kindlin-2 plays a key role in maintaining cardiac structure and normal heart function. The loss of kindlin-2 in adult CMs was associated with enlargement of the heart, and extensive fibrosis which eventuated in HF [78].

## 10. Focal Adhesion Kinase (FAK)

Focal adhesion kinase (FAK) is a non-receptor tyrosine kinase expressed widely, and has been implicated as an important mediator of integrin mediated transduction. Further, FAK has been demonstrated through early studies to be activated by the ECM or growth factors. Importantly, tyrosine phosphorylation of FAK has been associated with the formation of focal contacts. The FAK 4.1 protein ezrin radixin moesin (FERM) domain is important for the binding of integrins as well as the activation of integrin-FAK mediated non-receptor tyrosine kinases such as ETK [79]. The colocalization of FAK and integrins is an indirect association through binding to other integrin-related proteins like paxillin and talin [80,81] (Figure 3). Further, the mechanical activation of FAK has been demonstrated in vivo and in vitro [82,83]. FAK is implicated in myofibroblast transformation and collagen deposition [84,85,86,87]. Inhibition of FAK in cardiomyopathy leads to reduction in collagen I production and ⍺-SMA as well as, reduced ERK and AKT phosphorylation [88].

## 11. Integrin-Linked Kinase (ILK)

Integrin Linked kinase (ILK) is a protein kinase linking the cytoskeleton and mediating integrin signaling. It has been shown to bind to the cytoplasmic tails of β1 and β3 integrins [89,90]. ILK is thought to elicit its downstream biological activity through its role as a scaffolding protein, by binding other scaffolding proteins including PINCH, parvin and paxillin [91]. Parvin has been demonstrated to bind to the actin cytoskeleton [92] and PINCH binding can result in the initiation of the catalytic activity of ILK [91] (Figure 3). ILK is important to maintain proper myocyte architecture, as deletion of ILK in mouse hearts disrupts myocyte morphology leading to cardiac fibrosis [93].

## 12. Integrin Mediated Signaling

The mechano-sensing of integrins in response to cardiac injury, pressure or volume overload ultimately results in the activation of several downstream pathways, which mediate the activation of myofibroblasts and collagen production. One prominent pathway associated with integrin activation as a result of receptor ligand binding is TGF-β1. This mechanism, involving Small Mothers Against Decapentaplegic (SMADS) has been described in great depth [6,17,84,94,95,96].

The activation of integrins has been demonstrated to recruit Rho guanine nucleotide-exchange factors, thus initiating the activation of the Rho/ROCK pathway [5]. The Hippo related transcription factors, YAP/TAZ, have been demonstrated to be activated by mechanical signaling, through integrins, leading to activation of Src kinases, Rho-GTPases, ILK, and ultimately YAP/TAZ phosphorylation [97]. Finally, Wnt signaling pathway has been implicated with integrin/FAK activation in response to a stiffening ECM [98].

## 13. Spatial and Temporal Distribution and Functions of Integrins in Heart during Disease

The heart is comprised of four chambers each possessing cell populations that have been postulated to be region specific. The cells within each chamber differ in origin, electrophysiological properties, and gene expression. Importantly, the function and mechano-stress micro-environments of each chamber is distinct, resulting in a different response to disease. As previously described, CFs within diseased chambers have significantly altered phenotypes compared to that of healthy chambers and are referred to as myofibroblasts. Myofibroblasts produce significant amounts of ECM proteins and are marked by increased expression of various proteins such as ⍺-smooth muscle actin (⍺-SMA), platelet derived growth factor receptor (PDGFR)-⍺, PDGFR-β and periostin. In this section, we will explore how integrins expression changes across the myocardium in response to cardiac disease models and explore how integrins mediate cardiac fibrosis.

## 14. Integrins Across the Left Ventricle

### 14.1. Acute/Ischemic Injury

Following cardiac injury including MI, fibrosis remains an important response in order to replace cells within the injured area and stabilize heart function [6]. Initially, the significant loss of CMs due to necrotic cell death leads to an inflammatory phase which initiates the migration of macrophages and CFs to the injured myocardium [99,100]. Once macrophages remove dead tissue and ECM fragments from the injured myocardium, local cytokines stimulate the transformation of CFs into myofibroblasts [101,102,103]. These myofibroblasts secrete collagen to replace the lost cells, stabilizing the remaining tissue within the myocardium preventing rupture and death. However, once the scar has matured, myofibroblasts remaining in the infarcted area continue to secrete collagen type I and replace collagen type III [104]. Collagen type I within the infarct and per-infarct area undergoes cross linking by LOX iso forms [105]. Cross-linking of the collagen fibers leads to increased tensile strength and contraction of the infarct altering the ventricular geometry [10]. This results in LV dilation, as per Laplace’s law, LV dilatation increases local wall stress, further activating myofibroblasts in the infarct and remote area, further promoting deleterious remodeling, with infarct expansion and the production of fibrosis within the remote area. The remodeling leads to the development of HF, an increased chance of cardiac arrythmias and sudden cardiac death due to electrical inhomogeneity [106]. Since integrins are key mediators of ECM-cell attachment and mechano-sensing (LV wall stress) their expression and role following ischemic injury has been widely investigated.

Nawata et al. (1999) described the expression of ⍺1, ⍺3 and ⍺5 integrin in the rat heart following ligation of the left anterior descending (LAD) coronary artery [107]. This study demonstrated that ⍺5 integrin expression was increased mostly in the peri-infarct zone and remote areas early at 4 days and 7 days post ligation, followed by a decline to levels seen in control animals by 42-days post ligation [104]. Moreover, the integrin ⍺1 expression was significantly increased only in the peri-infarct zone and persisted until 42 days post ligation [107]. Modification of integrin expression correlated with the expression profiles of collagen and fibronectin post MI within the peri-infarct zone. The increase in ⍺5 integrin correlated with fibronectin expression and the increased ⍺1 integrin correlated with collagen expression, suggesting that integrins modify ECM expression. Krishnamurthy et al., showed that the heterozygous deletion of β1 integrin increased myocardial dysfunction with increased LV dilatation post MI [108]. This was associated with greater degrees of myocyte hypertrophy and apoptosis [108]. While there was no significant increase in fibrosis following LAD ligation, a trend to increased fibrosis was noted. Further Investigation by Sun et al. (2003), observed that following MI, integrins β1 and β3 gene expression was significantly upregulated at the site of the infarct [109]. To understand the β1 integrin expression further, they sought to understand the different isoforms β1D and β1A [109]. β1D expression was down regulated following MI, which was associated with upregulation of tumor necrosis factor ⍺ [109]. Human studies have described similar effects when samples from ischemic HF (IHF) patients who underwent transplant were evaluated [110]. The expression of integrin β1D was seen to be significantly lower in those patients suffering from IHF compared to that of control hearts [110]. Okada et al. (2013) sought to investigate the importance of integrins in ischemic damage following ischemic reperfusion injury of the left ventricle (LV) [68]. Using an overexpression model, Okada and colleagues demonstrated the importance of ⍺7 integrin, displaying that ⍺7 overexpression reduced the size of the infarct [68]. To further investigate the importance of integrins, they deleted the β1 integrin using a Cre with a myosin heavy chain promoter, which significantly increased the infarct size despite a similar area’s at risk [68]. Finally, Konstandin and colleagues have demonstrated a protective role ⍺5β1 integrin induced by fibronectin binding following MI [111]. Fibronectin KO mice had significantly increased infarct size and worsened function following MI due to attenuated cardiac progenitor cell (CPC) protection and proliferation. β1 and ⍺5 integrin knockdowns in CPC’s resulted in a blunted fibronectin induced CPC protection. Further, fibronectin treatment induces Pim 1 expression and overexpression of Pim 1 improved CPC protection which, was blunted by β1 integrin knockdown. Finally, FAK inhibition blocked fibronectin induced Pim 1 expression. Therefore, Konstandin and colleagues suggest fibronectin binding to ⍺5β1 induces protection and proliferation of CPC’s via activation of FAK and Pim 1 [111]. These studies have shown an important protective role for β1 integrin in regulating infarct formation. Further investigation of the contribution of ECM ligand binding, ECM production induced by integrins, and the downstream modulators is needed to understand how integrins modulate short-term infarct formation, and the long-term deleterious remodeling seen post infarct.

### 14.2. Pressure Overload

In response to PO the heart remodels, resulting in cardiac fibrosis and hypertrophy, in an attempt to normalize the increased wall stress. Fibrosis increases cardiac stiffness, which may manifest as diastolic dysfunction, and is associated with activation of cardiac fibroblasts to myofibroblasts, with the production of excessive ECM proteins [6,112]. Burges et al. sort to identify the effects of hypertension (coarctation of the abdominal aorta) and exercise training (XTR) upon integrin expression in the LV. They demonstrated that hypertension reduced α1, α2 and α5 expression, while increasing β1 integrin expression, compared to control [113]. Conversely, XTR reduced α1 and increased both α5 and β1 integrin expression, demonstrating the differential role of integrins in (patho)physiology and response to different forms of cardiac stress [113]. Moreover, Babbitt et al. investigated integrin expression and signaling following transverse aortic constriction (TAC). They demonstrated that TAC increased α1, α5, and β1 integrin mRNA as well as, α7 and β1D protein expression after 7 days [83]. Finally, FAK recruitment as measured via phosphorylated FAK which, was significantly increased 60 min post TAC [83]. The studies conducted by Burgess and Babbbit et al., demonstrate differential expression of integrins in response to pressure, but not as to how this affects ECM production and remodeling, which is an important next step. A study by Krishnamurthy et al. demonstrated the role of β1 integrin in myocardial remodeling after β—adrenergic stimulation [114]. Here, Krishnamurthy and colleagues, showed that heterozygous β1 integrin KO resulted in a blunted hypertrophic and fibrotic response in the LV, following β—adrenergic stimulation. A blunted fibrotic response coincided with increased matrix metalloproteinases (MMP)-2 protein levels 7- and 28-days post stimulation. Interestingly 7 days post stimulation, MMP-9 protein expression was increased in WT, but reduced in heterozygous β1 integrin KO. These changes in ECM molecules coincide with increased phosphorylated JNK and ERK protein expression [114]. Shai et al. inactivated β1 integrin exclusively in ventricular cardiac myocytes using a Cre-loxP strategy, and showed that Cre positive mice had approximately 18% β1D expression of controls, and upon reaching adulthood they developed “patchy” fibrosis across the myocardium. These results coincided with abnormal organization of integrin β1D and talin, showing a role for talin in β1D induced mechano-sensing. Finally, Cre positive mice did not tolerate TAC PO, as they had significantly worse survival (53% vs. 88%) [115]. The role of β1 integrin in PO described by the previous studies suggests it plays a significant role in ventricular remodeling and fibrosis. The knowledge that β1 pairs with ⍺ chain collagen binding integrins may suggest that this process is managed through collagen binding. Balasubramanian et al. investigated the role of β3 integrin in cardiac fibrosis, secondary to TAC. β3/2 integrin null mice, in response to TAC, displayed reduced accumulation of interstitial fibronectin and total collagen volume. Moreover, in order to study the impaired response of ECM accumulation in β3/2 integrin null mice, cardiac fibroblasts were analyzed from WT and β3/2 integrin null mice. β3/2 integrin null cardiac fibroblasts displayed a reduction in cell-matrix adhesion, spreading, migration, as well as reduced expression of the myofibroblast marker PDGFR. These results suggest a role for β3 integrin in myofibroblast transformation and cardiac fibrosis in response to TAC [28]. Finally, investigation of integrins in cardiac fibrosis induced by hypertension was investigated in spontaneously hypertensive rats (SHR), and compared to control Wistar rats [116]. SHR’s had significantly increased collagen I and TGF-β1 expression, which coincided with increased ⍺vβ5 integrin expression in both cardiac tissue and CFs [116]. SMAD2/3 signaling activation and ⍺-SMA was significantly higher in isolated SHR-CFs, following TGF-β1 treatment, an effect that was seen to be attenuated following inhibition of ⍺vβ5, which coincided with an attenuation of collagen I production [116]. As the β3 and ⍺vβ5 are RGD binding integrins, likely their role in cardiac fibrosis is via the activation of signaling molecules such as TGF-β after binding with ligands, such as fibronectin rather than collagen binding.

### 14.3. Diabetic Cardiomyopathy

Many patients with HF suffer from diabetes mellitus, and fibrosis is a frequent complication of diabetic cardiomyopathy (DCM), often referred to as the “frequent, forgotten and fatal complication of diabetes” [3,117,118,119]. DCM is initially characterized by myocardial fibrosis and dysfunctional remodeling, which is seen to be the production of disorganized fibrotic matrix.

Our group investigated the role of ⍺11 integrin in diabetes, where streptozotocin (STZ)-treated Sprague Dawley rats were found to have increased ⍺11 integrin mRNA and protein in isolated CFs compared to controls [64]. To further elucidate the mechanisms behind the excess fibrosis, siRNA Knockdown (KD) of α11 integrin was used in human fibroblasts plated on methylglyoxal-treated collagen. The KD of α11 integrin blocked the increase in TGFβ2 and α-SMA protein expression [64], suggesting that α11 integrin may mediate CF transformation through TGFβ2 and fibrosis in the diabetic heart. Further research by our group has demonstrated that glycated collagen binding to α11 integrin increased TGF-β2 expression, which coincided with the activation of α11 promoter. Interrogation of the α11 promoter demonstrated a Smad2/3 binding element [120]. Lastly, to further understand the role of α11 integrin in fibrosis observed in DCM, our lab treated α11-KO mice with STZ to induce diabetes. The loss of integrin α11 integrin reduced ECM production (collagen type I) and cardiac fibrosis compared to controls, but did not improve diastolic function [27]. The evidence from these studies suggests α11integrin is a master regulator of a “toxic triad” whereby, α11 binds to collagen resulting in TGF β signaling. The increase in collagen production, along with increased α11 expression results in a positive feedback loop, where α11 integrin acts to potentiate adverse cardiac remodeling and cardiac dysfunction.

The complexity of integrin binding within the LV displays that numerous questions remain unanswered. A role for the collagen binding integrins ⍺1, ⍺2 and ⍺11 has been demonstrated, but mechanisms are not well defined. The catalytic activity of integrins is managed by integrin related kinases. Understanding, how integrin-ligand binding modulates signaling mechanisms and the transformation of CFs to myofibroblasts is relevant. A differential integrin response was observed between the hypertension model and MI. This is likely dependent on disease etiology and progression, displaying the importance of understanding the roles of integrins, both in early remodeling as seen in MI, and the later fibrotic response known to cause LV dilation and HF.

## 15. Integrins in Right Ventricular Pressure Overload

Relative to its left-sided counterpart, the pathophysiological mechanisms underlying disease of the right ventricle (RV) remain ill-defined. This has been attributed to the incorrect belief that the RV is a passive structure, serving only to provide blood to the pulmonary circulation. With growing knowledge on the crucial interdependence between ventricles, as well as the RV’s prognostic significance in pulmonary hypertension (PH), including PH caused by left-HF, the RV has been receiving increased attention in the field of cardiac and cardiopulmonary research. The LV and RV differ in their embryological origin, structure, and CM architecture [121]. Interestingly, the RV has higher collagen content compared to the LV; however, the cause of this difference remains undetermined. The response of the RV to PO shares features with that of the LV, including initial compensatory hypertrophy which eventually decompensates and transitions into chamber dilation, activation of the fetal gene program, and fibrosis [122]. Dissimilarities also exist however, and are a topic of current research. A study by Urashima and colleagues demonstrated that the gene activation in response to PO shares much overlap but also differs between the ventricles, such as a heightened activation of Wnt signaling, Dickkopf 3, and lysyl oxidase in the RV compared to the LV [123]. It is likely that differences at the molecular level underly the responsiveness of the failing RV to various therapeutics conventionally used for the LV; however, this has not yet been explored in depth and remains one of the key questions in RV research.

Our knowledge of the roles that collagen-binding integrins play in the RV under PO remains limited. The β3 integrin was shown in early work by Kuppuswamy and colleagues, to be associated with cytoskeletal-Src, and FAK upon PO to the feline RV [124]. Although further research of the β3 integrin in KO models has implicated it as a key player in both cardiac hypertrophy and fibrosis, its known association with the non-collagen-binding αV integrin suggests that the mechanism by which it regulates these processes does not have a basis in direct integrin-collagen binding [28,125]. More recent work however has demonstrated a more prominent role for collagen-binding integrins in RV remodeling. In a rat model of RV dysfunction induced by pulmonary artery banding, our group demonstrated that α1β1 and α11β1 were upregulated in the both the RV and LV; including the α1β1A and α1β1D isoforms. These changes were accompanied by an increase in collagen type I, αSMA, TGFβ1, phospho-FAK and phospho-Smad2/3. To further investigate these signaling pathways at a cellular level, we subjected cultured RV fibroblasts to cyclical stretching to mimic the mechanical stress of PO. Cell stretching predictably induced the upregulation of profibrotic genes including αSMA, TGFβ1, and CTGF, as well as the β1A integrin. Inhibition of integrin α2β1 by BTT-3033 abrogated this stretch-induced response suggesting a key role for integrins in myofibroblast transformation in RV fibroblasts [126]. Despite the value of our study in establishing a clear association between collagen-binding integrin activity and profibrotic gene expression specifically in the mechanically stressed RV fibroblast as well as upregulation of α1β1 and α11β1 integrins in RV PO, further work is required to elucidate a molecular mechanism that causally connects collagen binding-mediated integrin signaling, to gene regulatory activity in the RV.

**Figure 4:**
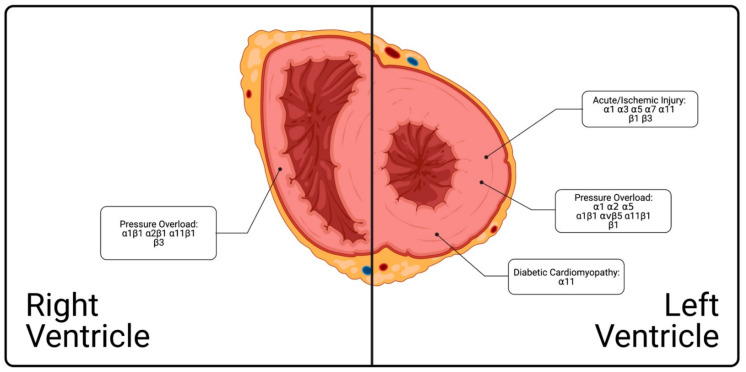
Summary of the current understanding of integrin involvement in left ventricular and right ventricular cardiac fibrosis. In the right ventricle, integrins α1β1, α2β1, α11β1, and β3 have shown modified expression under pressure overload conditions. In the left ventricle, integrins α1, α3, α5, α7, α11, β1, and β3 have shown modified expression in acute/ischemic injury; α1, α2, α5, α1β1, αvβ5, α11β1, and β1 in pressure overload; and α11 in diabetic cardiomyopathy.

Since our study only demonstrated the upregulation of α1 and α11 integrins in the pressure-overloaded RV, future studies using KO models could provide further insight on the contribution of specific collagen-binding integrins to the remodeling response of the RV to PAB. Such studies would be extremely useful to the integrin field because collagen-binding integrins share functional overlap. The α10β1 and α1β1 integrins primarily bind collagen IV and VI, while the α2β1 integrin selectively binds collagen I much like α11β1 [127]. Deconvoluting how each of the collagen-binding integrins respond and contribute to cardiac remodeling would provide more specific signaling frameworks to potentially target for therapeutic purposes which, with respect to the scarcity of knowledge on targetable RV-specific pathways, may be of great value. Thus, knowing what role integrins play in remodeling RV ECM and via which mechanisms as well as, how this differs to the LV remain important questions to the field of integrin research (Figure 4).

## 16. Atrium

The expression of integrins in disease has been investigated in the ventricles however, integrin expression in the atriums in the setting of disease, has yet to be examined. Further, there has been little work to investigate the integrin regional differences between the atriums and ventricles. Recent work by Wiencierz et al. (2015) has sought to differentiate atria CMs from ventricular CMs via integrin expression and investigated integrins ⍺1, ⍺5 and ⍺6 [128]. They demonstrated that ⍺6 integrin expression was differential between atrial and ventricular CMs during development, and was not a transient feature of development [128]. In fact, the differential expression of ⍺6 integrin persisted into adult CMs. This observation provides evidence for the temporal differential expression of integrins across the heart, further demonstrating the need to understand integrin expression changes temporally, and modulates disease progression.

## 17. Future Directions

In order to understand the role of integrins across the heart, it’s important to understand the different conditions in which each integrin is exposed to across chambers. The RV is normally exposed to pressures from 4 to 25 mmHg. While the LV is exposed to far greater pressures between 10 and 120 mmHg, in a healthy individual. Clearly, the hemodynamic stress exposed to the cells in each chamber is different. Whilst subpopulations of cardiac fibroblast appear to exist in the heart, it is unclear if this is due to inherent differences in these CF populations from an embryologic perspective, or if the differences observed occur secondary to the response CF’s undergo as a result of the hemodynamic stress they are placed under. If we consider that integrins are important mechano-sensors it is likely that their expression and function changes in response to the various hemodynamic and other stress changes in order to maintain appropriate ECM structures, in an attempt to dissipate wall stress. Therefore, integrins may, in part, be responsible for the observed differences in CF sub-populations.

The isolation of collagen producing fibroblast or myofibroblasts is difficult and the markers of the myofibroblast remain a contentious issue within the literature as most markers such as ⍺-SMA are inconsistent [129]. However, recent studies have identified markers that maybe more appropriate to mark CF or myofibroblasts including periostin and PDGFR-⍺ [130,131,132]. Further research is required to characterize these CF subpopulations, and whether integrins are key determinants of the response of these subpopulations.

## 18. Conclusions

The expressions and function of integrins plays a critical role in both cardiac health and disease, by providing a key link between the CM, ECM and CF. However, the precise understanding of expression, control and regulation, in both a temporal and spatial dimension requires further research. Understanding, how integrins modulate myofibroblast transformation, and their relationship to ECM production requires further elucidation. Likewise, understanding the cellular subpopulations, and what drives this difference may help further the discovery of novel CM–CF interactions, where integrins play an essential role.
